# Sub-Lethal Effects of Pirimiphos-Methyl Are Expressed to Different Levels in Wings of Three Stored-Product Coleopterans: A Geometric Morphometrics Investigation

**DOI:** 10.3390/insects14050430

**Published:** 2023-04-30

**Authors:** Maria C. Boukouvala, Nickolas G. Kavallieratos, Vladimir Žikić, Saša S. Stanković, Marijana Ilić Milošević, Anna Skourti, Maja Lazarević

**Affiliations:** 1 Laboratory of Agricultural Zoology and Entomology, Department of Crop Science, Agricultural University of Athens, 75 Iera Odos str., 11855 Athens, Greece; 2 Department of Biology and Ecology, Faculty of Sciences and Mathematics, University of Niš, Višegradska 33, 18000 Niš, Serbia

**Keywords:** elytra, hindwings, morphometry, pesticide, Tenebrionidae, Bostrychidae

## Abstract

**Simple Summary:**

We used the geometric morphometrics method to explore whether the exposure of the parental females to pirimiphos-methyl at short exposure times may impact the wings of the F_1_ generation of *Rhyzopertha dominica*, *Prostephanus truncatus*, and *Tenebrio molitor*. For this purpose, adult females of the three coleopterans were exposed to treated concrete at the label dose for 30 min, 3, 5, 8, 16, 24, and 36 h. Their offspring responded differently to pirimiphos-methyl. The most noticeable deformations were observed in the elytra and hindwings of *T. molitor*, and males experienced deeper morphological changes than females. The hindwings of *P. truncatus* exhibited morphological deformations after 36 h of exposure to pirimiphos-methyl. *Rhyzopertha dominica* proved the least susceptible species since pirimiphos-methyl did not have any effect on both elytra and hindwings.

**Abstract:**

*Tenebrio molitor* L. (Coleoptera: Tenebrionidae), *Prostephanus truncatus* (Horn), and *Rhyzopertha dominica* (F.) (Coleoptera: Bostrychidae) are noxious insect pests of grains in storages. Pirimiphos-methyl is widely used to protect grains at the post-harvest stage. However, the sub-lethal impact of this active ingredient on the offspring of all three coleopterans remains unknown. Thus, mated females of each species were exposed separately to pirimiphos-methyl at short exposures (30 min, 3, 5, 8, 16, 24, and 36 h), where the elytra and hindwings of the adult progeny were analyzed with the geometric morphometrics method. Males and females of all species were incorporated into the analysis. The results revealed variability among species. *Tenebrio molitor* was the most sensitive among three species, displaying significant deformations in the elytra and hindwings. Males had more conspicuous morphological changes than females. *Prostephanus truncatus* hindwings exhibited deformities after 36 h of exposure to pirimiphos-methyl. In contrast, *R. dominica* offspring were not affected by pirimiphos-methyl. In light of our findings, organophosphorus insecticides may cause variable sub-lethal effects to stored-product insects. This issue may lead to different insecticidal treatments according to the targeted stored-product species.

## 1. Introduction

Coleoptera, including approximately 400,000 described species [1,2], is a highly important insect group for agriculture and forestry [3]. Several coleopteran species, such as species of the families Tenebrionidae and Bostrychidae, are important pests in food storage or processing facilities [4]. *Tenebrio molitor* L. (Coleoptera: Tenebrionidae) is a pest of stored commodities worldwide [5], infesting a wide spectrum of products of animal and plant origin [6]. Furthermore, it can be hazardous to humans due to allergies caused by the contamination of commodities from the secretion of quinones [7,8]. *Prostephanus truncatus* (Horn) (Coleoptera: Bostrychidae) causes extensive and irreparable damages to stored dried tubers of cassava and maize [9,10]. It was accidentally introduced to Africa from Central America in the late 1970s [11]. The level of damage to stored maize is greater than the damage caused by other harmful insects, such as *Sitophilus zeamais* Motschulsky (Coleoptera: Curculionidae) and *Rhyzopertha dominica* (F.) (Coleoptera: Bostrychidae) [12,13]. *Rhyzopertha dominica* is a global insect pest of Indian origin [14,15]. This polyphagous species damages numerous stored-product commodities (e.g., cereal grains, cocoa beans, cassava, biscuits, birdseed, beans, dried fruits, nuts, tobacco, spices, peanuts) [16,17]. The feeding activity of *R. dominica* larvae and adults causes severe seed damage (e.g., degradation of quality, change in the chemical and physical composition) resulting in economic losses [18,19,20]. 

Pirimiphos-methyl, an organophosphorus insecticide, is used against a wide spectrum of stored-product pests worldwide [21,22,23,24]. It is responsible for the phosphorylation of the acetylcholinesterase (AChE) that regulates the hydrolysis of acetylcholine in the synaptic cleft of the neural system of insects [25,26,27]. Several research efforts have revealed the high efficacy of pirimiphos-methyl against stored-product insects. For instance, wheat treated with this insecticide caused 100% mortality to adults of *Plodia interpunctella* (Hübner) (Lepidoptera: Pyralidae), *Tribolium castaneum* (Herbst) (Coleoptera: Tenebrionidae), *Sitophilus oryzae* (L.) (Coleoptera: Curculionidae), and *Cryptolestes ferrugineus* (Stephens) (Coleoptera: Laemophloeidae) [28]. Kavallieratos et al. [29] documented that 10 ppm of pirimiphos-methyl applied on paddy rice, barley, wheat, and maize caused 100% mortality in larvae and adults of *Trogoderma granarium* Everts (Coleoptera: Dermestidae). This insecticide suppressed the hatchability of the exposed eggs of *T. granarium* on concrete surfaces [24].

The application of insecticides (e.g., neonicotinoids, organophosphorus, spinosyns) to the immature stages of insects may have a sub-lethal impact on the adult stage by affecting various biological traits, such as fertility and fecundity, or may lead to deformations of certain parts of their bodies [30,31,32,33,34,35,36]. Sub-lethal effects have been also reported in the F_1_ generation, following the exposure of the parental individuals to a toxic agent [37,38,39]. For instance, the net reproductive rate (*R*_0_) and fecundity of the F_1_ generation of *Aphis gossypii* (Glover) (Hemiptera: Aphididae), emerged from parental individuals that were treated with the LC_25_ of flupyradifurone, were lower in comparison with the control group [40]. Similarly, the reproductive capacity of *T. castaneum* progeny derived from parental females exposed to the label dose of pirimiphos-methyl for 24 or 72 h was significantly reduced when compared to control beetles [37]. One other recent study revealed that the toxicity of pirimiphos-methyl to parental adult females of *T. granarium* resulted in malformed offspring [41]. Based on geometric morphometrics, the authors reported that the elytra and hindwings of the F_1_ generation underwent certain changes in their original form that were more noticeable on the hindwings [41]. This method has been widely used to detect minor distortions in the morphology of wings and other anatomical structures of insects [42,43]. However, it is unknown whether pirimiphos-methyl causes wing deformities to stored-product tenebrionids and bostrychids, emerging from treated parental adults. Thus, the objective of the current research effort was to investigate the changes in the morphology of elytra and hindwings of the F_1_ generation of *T. molitor*, *R. dominica*, and *P. truncatus*, whose parents had been exposed to pirimiphos-methyl-treated concrete surfaces under different intervals, using the geometric morphometrics method. This study points out the necessity of designing pest management strategies on the basis of the presence of the targeted stored-product insect species. 

## 2. Materials and Methods

### 2.1. Insects

The parental individuals of the three species were taken from cultures maintained in the insectary of the Laboratory of Agricultural Zoology and Entomology (Agricultural University of Athens) since 2014. *Tenebrio molitor* was kept on oat bran containing potato slices that served as sources of moisture [44]. *Rhyzopertha dominica* and *P. truncatus* were cultured on intact hard wheat and maize, respectively. All colonies were maintained at 30 °C, 65% relative humidity (RH), and in 0:24 L:D (light–dark cycle). The age of *T. molitor* and *P. truncatus* females was >5 days old since copulation occurs 5 days after eclosion for *T. molitor* [45,46], while *P. truncatus* starts laying eggs 5 to 10 days after the emergence of adults [47]. The age of *R. dominica* females was >24 h old because this species is able to mate within the first day of eclosion [48]. The age of adults of all 3 species used in the experiments did not exceed 15 days old.

### 2.2. Insecticide Bioassays

The commercial formulation Actellic EC (Syngenta, Anthousa, Greece), a microencapsulated formulation that contains 50% pirimiphos-methyl active ingredient (a.i.), was utilized in the experiment. Pirimiphos-methyl was tested at 0.05 mg a.i./cm^2^, i.e., the recommended dose for treatments on surfaces. The preparation of the concrete surfaces was carried out 24 h before the start of the experiments. A layer of cement CEM I 52.5 N (Durostick, Aspropyrgos, Greece) was created on the bottom of 630 Petri dishes (surface area 50.27 cm^2^). Then, the upper parts of the internal walls of the dishes were coated with polytetrafluoroethylene (60 wt% dispersion in H_2_O) (Sigma-Aldrich Chemie GmbH, Taufkirchen, Germany) to prevent the beetles from walking out. An aqueous solution with the appropriate concentration of the label dose of pirimiphos-methyl was prepared. Then, 1 mL was taken to spray each dish separately with a BD-134K airbrush (Fengda, Yorkshire, UK). An additional set of 210 dishes (10 dishes × 7 exposures × 3 insect species), covered with cement, were sprayed with water (controls) using one other airbrush that was exclusively reserved to treat controls. The females of the three species were allowed to mate for a period of 3 days. Next, 30 female adults of *T. molitor*, *R. dominica*, or *P. truncatus* were put separately into each dish and transferred at 30 °C, 65% RH into incubators, and in a total absence of light for 0 (control), 30 min, 3, 5, 8, 16, 24, and 36 h. After the lapse of the aforementioned intervals, the females of *R. dominica* and *P. truncatus* were placed separately (3 replicates, each constituted by 10 dishes [49]) in vials made of glass (height: 12.5 cm, diameter: 7.5 cm), each containing 30 g kernels of hard wheat or maize, respectively. Females of *T. molitor* were put in 8 glass jars, each containing the surviving females after their initial exposure to pirimiphos-methyl, with a capacity of 3 L each with a substrate consisting of 300 g oat bran and potato slices. Subsequently, all vials and jars were kept in the same conditions for 14 days. This interval is proper to receive satisfactory F_1_ progeny [50]. After this interval, the parental adult individuals of *T. molitor*, *P. truncatus*, and *R. dominica* were taken out from the vials using the US standard testing sieves No. 8 (2.36 mm openings), No. 10 (2 mm openings), and No. 12 (1.7 mm openings) (Advantech Manufacturing, Inc., New Berlin, WI, USA), respectively. Then, the jars were returned back to the incubators for a period of 45, 60, or 65 days for *R. dominica*, *P. truncatus*, and *Τ. molitor*, respectively [51,52,53], to obtain the adult progeny. The sex of *T. molitor*, *R. dominica*, and *P. truncatus* adults was determined according to Crombie [54], Shires and McCarthy [55], and Bhattacharya et al. [56], respectively. Then, all sexed individuals per species and exposure interval were preserved at 96% ethyl alcohol until geometric morphometrics analysis.

### 2.3. Wing Dissection, Preparation, and Taking Photos

Fifteen F_1_ adults of both sexes of all three coleopteran species were selected for analysis. Control and pirimiphos-methyl-treated individuals were rehydrated in distilled water. Specimens were placed separately in 2 mL thermostable plastic tubes filled with distilled water and boiled for an interval of 15 min at 100 °C in a laboratory glass beaker (Sigma-Aldrich Chemie GmbH, Taufkirchen, Germany) with a volume of 100 mL. Then, the right elytron and hindwing were dissected with fine forceps on small plastic trays. The dissection process was conducted using a stereomicroscope Zeiss Discovery V8 (Carl Zeiss MicroImaging GmbH, Göttingen, Germany) at a zoom ratio of 8 and total magnification × 10.0–80.0. Elytra were directly glued with Berlese medium to microscope slides. The hindwings were covered with a coverslip to be pressed and brought into a 2D position. Elytra and hindwings were photographed with a Leica DFC490 camera (Leica Microsystems, Wetzlar, Germany), adapted to a microscope Leica 2500 (Leica Microsystems, Wetzlar, Germany), at 5.0–20.0× total magnification.

### 2.4. Wing Digitalization and Geometric Morphometrics

**Figure 1:**
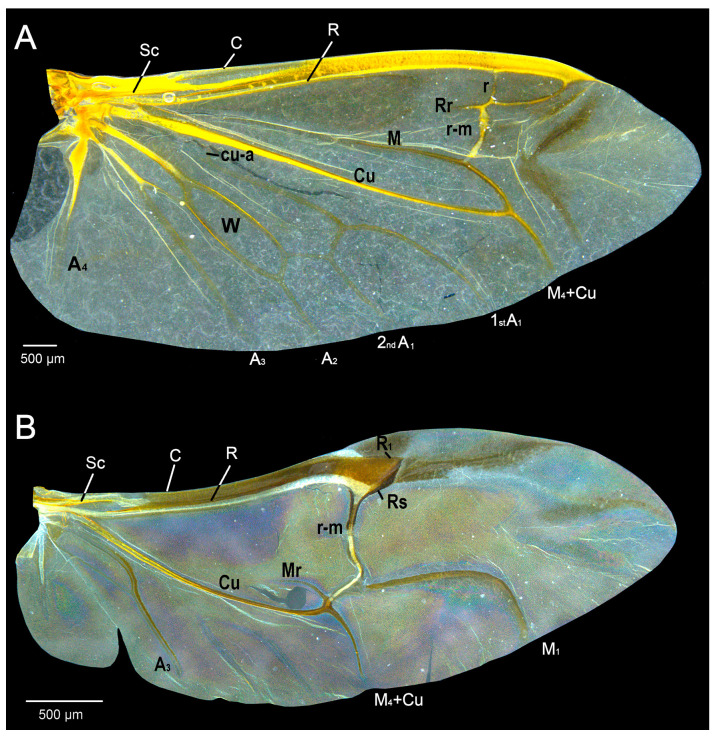
Hindwing venation nomenclature. (**A**)—*Tenebrio molitor*, (**B**)—*Prostephanus truncatus*. Wing vein abbreviations: (A) = anal, (Cu) = cubital, (C) = costal, (M) = medial, (R) = radial, (RS) = radial sector, (r-m) = radio-medial, (Sc) = subcostal, (W) = wedge-cell [57]. Numbers indicate vein branches.

**Figure 2:**
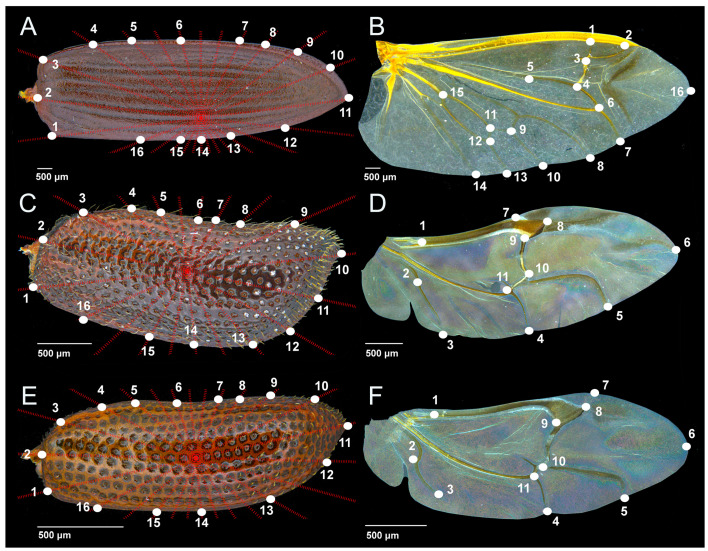
Positions of landmarks. *Tenebrio molitor*: (**A**)—elytron, (**B**)—hindwing; *Prostephanus truncatus*: (**C**)—elytron, (**D**)—hindwing; *Rhyzoperta dominica* (**E**)—elytron, (**F**)—hindwing. Selected landmarks are labeled with numbers. Dashed red lines are auxiliary lines for semi-landmarks positioning.

The selection and placement of landmarks (LM) on the hindwings of the three investigated species is determined by wing venation (Figure 1). Since the hindwings of *P. truncatus* and *R. dominica* have very similar venation (Figure 1B and Figure 2D,F), we chose *P. truncatus* as a model to present and mark the veins. The hindwings of *T. molitor* are complex in structure (Figure 1A and Figure 2B), with highly developed venation; thus, the selection of landmarks is different compared to *R. dominica* and *P. truncatus* (Figure 1B and Figure 2D,F). It should be noted here that in addition to the differences in wing venation, *T. molitor* is more than twice as large as *P. truncatus* and *R. dominica* (see scale bars below the wings in Figure 1 and Figure 2). The position of the landmarks on the hindwings was determined after examining the entire sample (control sample and pesticide-treated samples). We selected the junction or the ending of the hindwing vein present in each specimen so that the position and the number of landmarks were the same in the control group as in the pesticide-exposed groups.

The method of geometric morphometrics [58] is based on the analysis of a set of selected landmarks by which morphologically similar structures are compared. In addition to the visualization of the analyzed samples in the morphospace defined in two or three dimensions, the changes in the size and shape of the structures can be monitored via deformation grids. The forewings are contoured with positioning true (LM) and semi-landmarks (S-LM), while the hindwings are defined only with true landmarks. True LMs were directly placed using tpsDig2 software [59], used for both elytra and hindwings. Auxiliary lines in the form of the star were made for elytra using the MakeFan6 software [60]. Afterwards, the points were auxiliary lines crossing the edge of the forewings and semi-landmarks were placed using the tpsDig2 software. 

To calculate shape coordinates (Procrustes coordinates), we used the Generalized Procrustes Analysis (GPA). LM configurations were scaled, translated, and rotated relative to consensus configuration [61,62]. Using MorphoJ software [63] for each set of LMs, the wing size was calculated via centroid size (CS).

### 2.5. Statistical Analysis

To test for size differences in the elytra and hindwings, we provided an analysis of variance (ANOVA). To compare wings, the mean value for each wing was calculated as (CS). Centroid size was calculated for both sexes of each of the three species for both wing types. Multivariate analysis of variance (MANOVA) was applied to determine differences in wing shape, where shape variables (Procrustes coordinates) are dependent variables. Both statistical analyses were conducted in R Studio software [64]. Independent variables represent a priori constructed groups (controls and treatments) for both sexes. A statistical test of the null hypothesis, i.e., the shape does not change after exposure to the insecticides, was conducted. The percentage and possible changes in wing shape were obtained using the MorphoJ software [63]. Additionally, to monitor the differences in the shape of the elytra and hindwings in morphospace, a canonical analysis of variance (CVA) and pairwise permutation test on Procrustes distances (distance of each landmark constellation from the central position) were performed using the same software. The results of the canonical variate analyses were exported from MorphoJ software and graphs were made using the ggplot2 software package implemented in R Studio software [65].

## 3. Results

The direct mortalities of *T. molitor*, *P. truncatus*, and *R. dominica* maternal individuals that had been exposed to concrete treated with pirimiphos-methyl at control, 30 min, 3, 5, 8, 16, 24, and 36 h are reported in Table 1. Furthermore, delayed mortalities of maternal adults of the tested species after a 14-days exposure to different commodities, following different exposures on concrete treated with pirimiphos-methyl, are shown in Table 2.

Analyzing the variability in the size of the elytra and hindwings in all three storage pests, differences in the size of the wings were statistically significant for all three species: *T. molitor*: for elytra (*F*_(15, 221)_ = 162.9, *p* < 0.001), for hindwings (*F*_(15, 224)_ = 20.99, *p* < 0.001); *P. truncatus*: for elytra (*F*_(15, 224)_ = 1.93, *p* = 0.021), for hindwings (*F*_(15, 219)_ = 3.11, *p* < 0.001); *R. dominica*: for elytra (*F*_(15, 224)_ = 1.753, *p* = 0.043), for hindwings (*F*_(15, 219)_ = 1.91, *p* = 0.002). Likewise, by examining the variability in wing shape, significant differences in elytra and hindwings were found in all three species: *T. molitor* elytra (Wilks’λ = 0.046, *F*_(405, 2618)_ = 1.702, *p* < 0.001) and hindwings (Wilks’λ = 0.029, *F*_(405, 2058)_ = 2.028, *p* < 0.001); *P. truncatus* elytra (Wilks’λ = 0.047, *F*_(405, 2675)_ = 1.714, *p* < 0.001) and hindwings (Wilks’λ = 0.058, *F*_(270, 2378)_ = 2.443, *p* < 0.001); *R. dominica* elytra (Wilks’λ = 0.047, *F*_(405, 2657)_ = 1.714, *p* < 0.001) and hindwings (Wilks’λ = 0.093, *F*_(270, 2378)_ = 2.009, *p* < 0.001).

The pairwise permutation tests conducted on the Procrustes distances revealed which groups showed statistically significant differences (Table S1). For all three species, significant differences were obtained for hindwings than for elytra (Table S1). Especially in the case of the hindwings of *T. molitor*, most groups/treatments showed between-group statistically significant differences (*p* < 0.05), except in 16 cases, as seen in the 8 h and 36 h treatments in males (Table S1). On the other hand, only twelve treatment pairs for elytra exhibited statistically significant differentiation in the permutation test. Fewer significant pairs were observed in *P. truncatus*, around 1/3 for hindwings, and less than 1/4 for elytra. The pairwise permutation tests in the case of elytra of *R. dominica* showed that only two pairs were significant (control female–30 min female, and 30 min female–16 h female), but half of the analyzed pairs showed significant differences when hindwings were analyzed (Table S1). 

**Figure 3:**
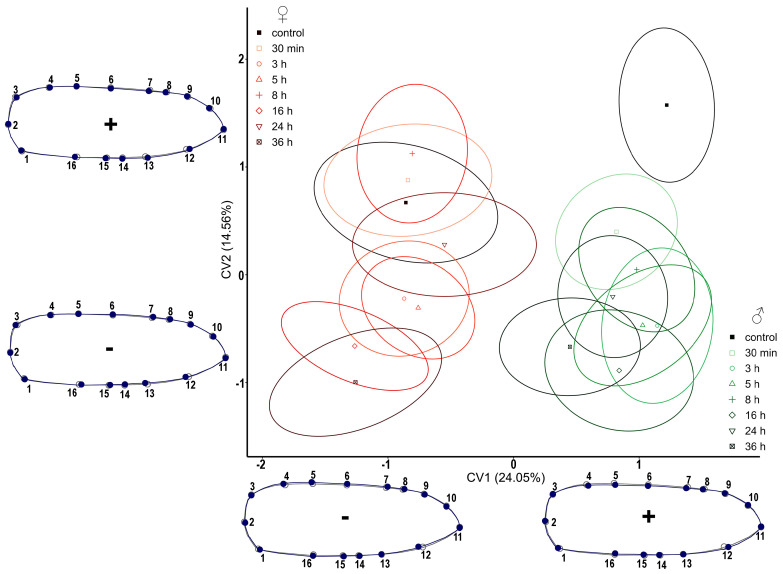
Scatterplot of *Tenebrio molitor* elytra in CVA morphospace. Shades of red ellipses mark the female groups; shades of green ellipses mark the male groups. Ellipses represent mean values (90% confidence interval). A set of landmarks marked with numbers describes the contour of the elytron. The grey line represents the shape of the mean value of elytron, while the blue line exemplifies the shape of the elytron for the minimal and maximal values (“−”and “+” signs, respectively) along the CV axes.

In the display of the elytra of *T. molitor*, morphological changes are more conspicuous in males than in females (Figure 3). *Tenebrio molitor* females have a continuous distribution of treatments and the control group. In addition to the sexual dimorphism, according to the shape of the elytra which is evident in the graphic, all groups of females are positioned in the negative part of the CV1 axis. Males are distributed in the positive part of the CV1 axis. Based on 16 LMs describing the elytra shape, the wings of males are narrower than those of females (CV1). After pirimiphos-methyl treatment, they became wider, which is confirmed by their grouping of treatments in the negative part of CV2.

**Figure 4:**
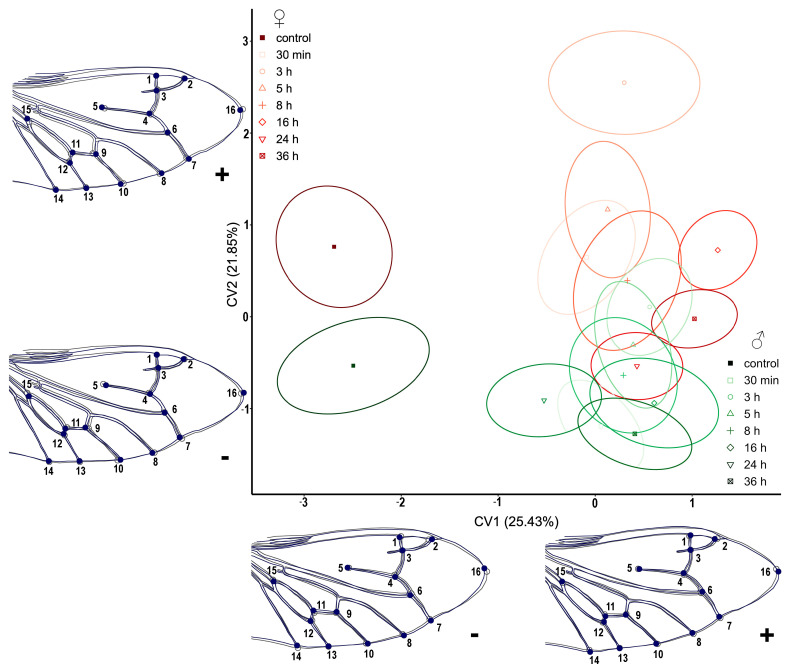
Scatterplot of *Tenebrio molitor* hindwings in CVA morphospace. Shades of red ellipses mark the female groups; shades of green ellipses mark the male groups. Ellipses represent mean values (90% confidence interval). A set of landmarks marked with numbers describes the contour of the hindwing. The grey line characterizes the shape of mean value for hindwings, while the blue line exemplifies the shape of the hindwings for the minimal and maximal value (“−” and “+” signs, respectively) along the CV axes.

The CVA analysis of the hindwings of *T. molitor* revealed that the separation of the treatment groups in both males and females is much more obvious than in the analysis of elytra (Figure 4). All treatments are positioned in the CV1+ section, while the control groups of both sexes are placed on the opposite side (CV1-). Similarly, the biggest differences in wing shape are noticeable mainly in the distal part, represented by LM2, LM7, and LM16. Following the changes via CV2, these landmarks are joined by LM1 and LM5. Sexual dimorphism is also exhibited by the division of males and females along the CV2 axis. For both treatments and control groups, males are positioned in the negative and females in the positive part (Figure 4).

**Figure 5:**
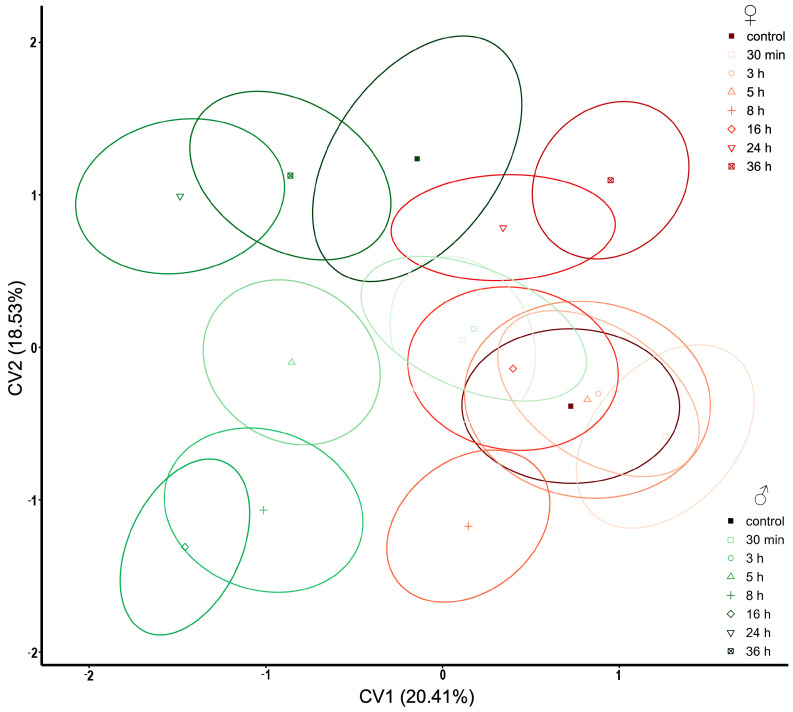
Scatterplot of *Prostephanus truncatus* elytra in CVA morphospace. Shades of red ellipses mark the female groups; shades of green ellipses mark the male groups. Ellipses represent mean values (90% confidence interval).

**Figure 6:**
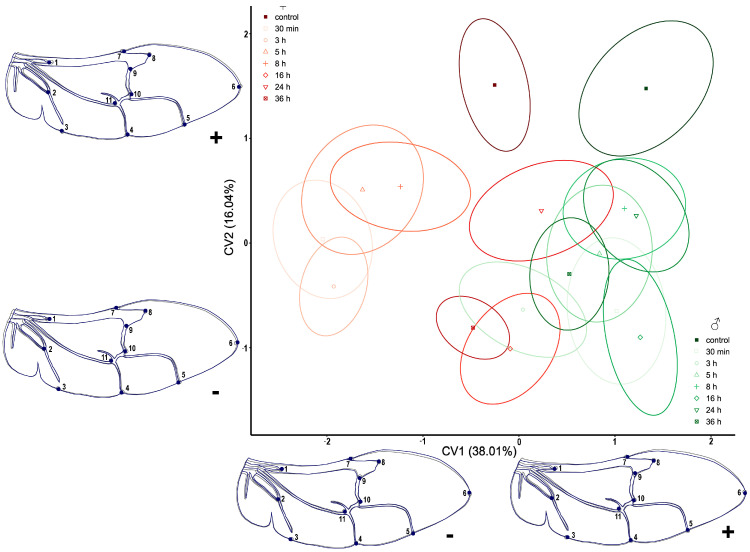
Scatterplot of *Prostephanus truncatus* hindwings in CVA morphospace. Shades of red ellipses mark the female groups; shades of green ellipses mark the male groups. Ellipses represent mean values (90% confidence interval). A set of landmarks marked with numbers describes the contour of the hindwing. The grey line characterizes the shape of the mean value of hindwings, while the blue line exemplifies the shape of the elytron for the minimal and maximal value (“−” and “+” signs, respectively) along the CV axes.

**Figure 7:**
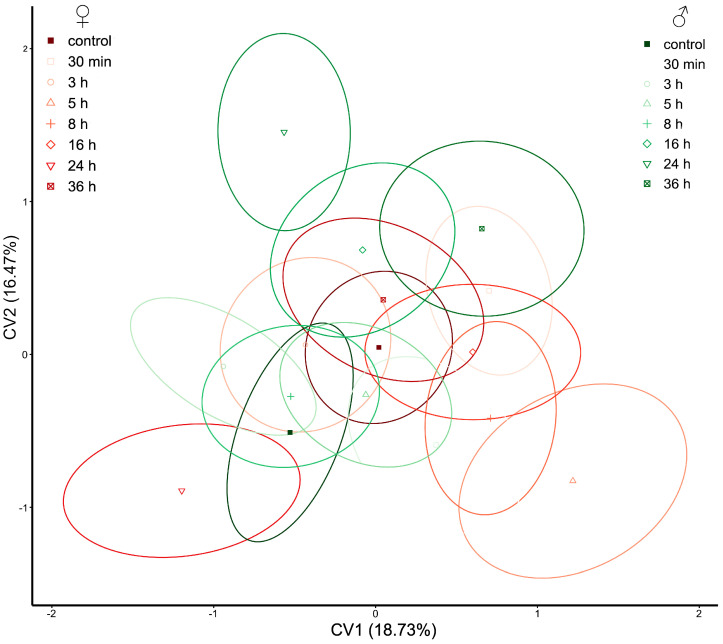
Scatterplot of *Rhyzopertha dominica* elytra in CVA morphospace. Shades of red ellipses mark the female groups; shades of green ellipses mark the male groups. Ellipses represent mean values (90% confidence interval).

**Figure 8:**
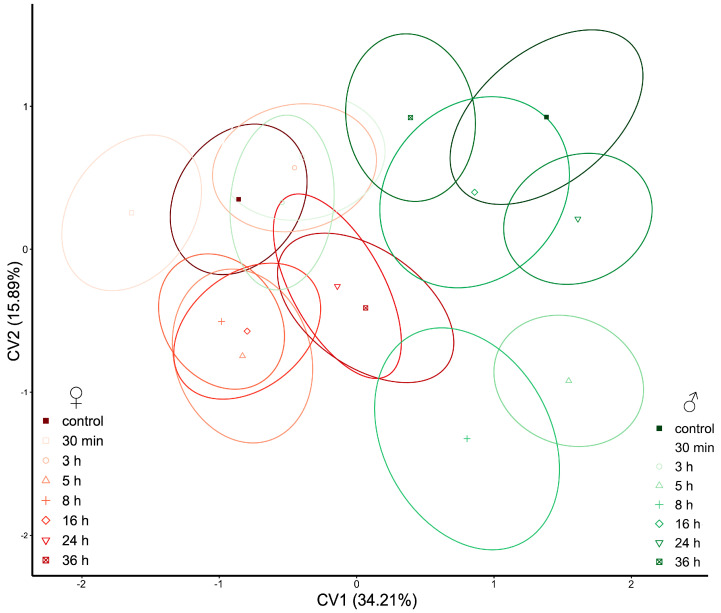
Scatterplot of *Rhyzopertha dominica* hindwings in CVA morphospace. Shades of red ellipses mark the female groups; shades of green ellipses mark the male groups. Ellipses represent mean values (90% confidence interval).

By visualizing the results in morphospace, comparing all six wing analyses of the three selected pests, the most noticeable shape changes in both pairs of wings occurred in *T. molitor* (Figure 3 and Figure 4). In cases where there was no between-group treatment separation, only their distribution in the form of ellipses in the CVA morphospace is shown, but not the wing shape changes themselves because they are negligible (Figure 5, Figure 6, Figure 7 and Figure 8).

Changes in hindwings in *P. truncatus* are very subtle. Treatments and controls for both sexes are mixed in CV1×CV2 morphospace (Figure 5).

The hindwings of *P. truncatus* are susceptible to pirimiphos-methyl, since the treated groups are separated from the controls by the CV2 axis (Figure 6). Both male and female control groups are in the CV2 positive part, while there is a tendency of displacement of the treated groups toward the negative part of the CV2. This is especially exhibited in the longest time treatment with pirimiphos-methyl, where the 36 h groups are the furthest from the controls (Figure 6).

Unlike the previous two species, *R. dominica* shows no effect on exposure to pirimiphos-methyl since all the treated groups and controls are scattered in the morphospace (Figure 7 and Figure 8).

## 4. Discussion

To the best of our knowledge, this is the first study of the sub-lethal effect of pirimiphos-methyl on the morphology of *T. molitor*, *R. dominica*, and *P. truncatus* offspring. Interestingly, the three selected species responded differently to pirimiphos-methyl, exhibiting variability in wing deformations. Concretely, the most susceptible species was *T. molitor*, which showed remarkable changes in the shape of both the elytra and hindwings, followed by *P. truncatus*, having the most noticeable changes in the hindwings after 36 h of exposure. *Rhyzopertha dominica* was by far the most tolerant species to pirimiphos-methyl since no significant differences were observed on its wings following all treatments, when compared with the control group. 

Pirimiphos-methyl has been previously evaluated against all three species, achieving a satisfactory level of control. For example, this insecticide was highly effective against *T. molitor* and *P. truncatus*. All adults of *P. truncatus* were killed 14 days after the initial exposure to maize sprayed with 4 ppm pirimiphos-methyl, at 20, 25, and 30 °C [66]. Similar results were obtained for *T. molitor* adults after the same exposure to barley, maize, and wheat sprayed with 5 ppm pirimiphos-methyl 14 days post-exposure [23]. Concerning *R. dominica*, 1.3 (out of 25) adults/container survived after a week of exposure to stored wheat sprayed with 8 ppm pirimiphos-methyl [28]. The application of pirimiphos-methyl at the label dose to polypropylene storage bags caused complete mortality to *P. truncatus* and *R. dominica* adults 5 days post-exposure [67]. All the above results indicate that pirimiphos-methyl performs equally in all tested species in terms of direct mortality, either as grain or surface treatments. Therefore, the revealed categorization of *T. molitor*, *P. truncatus*, and *R. dominica*, according to the degree of deformation, is not related to their susceptibility to pirimiphos-methyl. The insecticide likely affected the eggs produced by the treated females, at least in the case of *T. molitor* and *P. truncatus* given that only these species exhibited deformed wings on the basis of our findings. Previously, Uggini et al. [68] found that eggs treated with the organophosphate chlorpyrifos resulted in numeral skeletal anomalies in chicks due to the inhibition of acetylcholinesterase, which affects the normal activity of the neurotransmitter acetylcholine. This neurotransmitter activates the genes responsible for cell replication and subsequently cell differentiation during embryogenesis. Strong evidence of a similar effect of pirimiphos-methyl on insects’ wings has been provided by Lazarević et al. [41]. One other possible exegesis could be the different sizes of the three beetle species. Considering that the smaller-sized wings are less rigid in comparison to the bigger wings [69,70], the deformations caused by pirimiphos-methyl were more intensive in the larger *T. molitor* than in the smaller bostrychids. Indeed, *T. molitor*, which is about 20 mm long [71], was the most affected species, showing shape variability in both wings. In *P. truncatus*, which is about 4 mm in length [72], the hindwings were deformed by pirimiphos-methyl, whereas no effects of the insecticide were detected in the wings of the smallest (2–3 mm) in length *R. dominica* [50].

Our results for *T. molitor* stand in agreement with those related to *T. granarium* [41]. The latter species exhibited deformations in both elytra and hindwings, but the most noticeable changes were found in hindwings. This phenomenon could be explained by the fact that the structures of the elytra are less complex compared to the hindwings [73,74]. Based on this study and Lazarević et al. [41], it is confirmed that elytra, as a highly chitinized structure that primarily has a protective function, responded less to insecticidal treatments than hindwings. Lazarević et al. [41] reported a higher level of deformities in the wings of *T. granarium* females than in the wings of males. This can be explained by the different sizes of sexes. *Trogoderma granarium* females are 1.4 times larger than males [75]. In contrast, although both sexes are roughly the same size, wing shape changes were observed in males of *T. molitor* but not in females, indicating that males are more sensitive to the stressor regardless of the size of the sex. Whether this observation is a general characteristic among coleopterans whose sexes do not exhibit obvious differences in their sizes remains to be uncovered.

The exposure of insects to a toxic agent can adversely affect their fitness or offspring in different ways. For example, the topical application of 1 mL of the insect growth regulator lufenuron at different sub-lethal concentrations in fifth instar nymphs on two strains of *Cimex lectularius* (Hemiptera: Cimicidae) resulted in morphological abnormalities in the legs of the late instars and adults [76]. The abnormal legs negatively affected the walking activity of both strains. In our study, the observed deformities in the hindwings of *P. truncatus*, which can fly [50], may impact its flight activity, and thus its dispersal. In the case of *T. molitor*, the abnormal elytra may not properly protect the insect’s body, exposing it to environmental hazards. It is unknown whether the observed wing deformities of *T. molitor* alter the mating and laterality behavior which are crucial factors for the successful copulation of this species [77], an issue that should be clarified.

The results of the current study revealed that the exposure to pirimiphos-methyl at the label dose affected the wing morphology of *T. molitor* and *P. truncatus* offspring, whereas *R. dominica* progeny did not show any wing deformities. The fact that the followed concept of short exposures of maternal females led to deformed offspring is important, given that the contact of insects with the insecticide is not continuous in storage spaces [78,79]. Therefore, these females will lay eggs that will generate deformed adults. Treatments can be moderated for *T. molitor* (when it is considered as pest) and *P. truncatus* as they exhibited sensitivity to pirimiphos-methyl in the F_1_ generation. This current knowledge indicates the need to develop a separate treatment plan for the management of *R. dominica*. The sub-lethal effects of contact insecticides on stored-product insects represent a new approach towards their accurate control. Further research knowledge is needed to better understand the puzzling interactions of insects with insecticides. 

## Figures and Tables

**Table 1:** Mean direct mortality (% ± SE) of *Tenebrio molitor*, *Prostephanus truncatus*, and *Rhyzopertha dominica* maternal individuals exposed to concrete treated with pirimiphos-methyl at control, 30 min, 3, 5, 8, 16, 24, and 36 h.

	Species		
Exposure	*Tenebrio molitor*	*Prostephanus truncatus*	*Rhyzopertha dominica*
Control	0.0 ± 0.0	0.0 ± 0.0	0.0 ± 0.0
30 min	0.0 ± 0.0	0.0 ± 0.0	0.0 ± 0.0
3 h	0.0 ± 0.0	0.0 ± 0.0	0.0 ± 0.0
5 h	0.0 ± 0.0	0.0 ± 0.0	0.0 ± 0.0
8 h	0.0 ± 0.0	0.0 ± 0.0	0.0 ± 0.0
16 h	0.0 ± 0.0	0.0 ± 0.0	0.0 ± 0.0
24 h	10.0 ± 5.6	10.0 ± 5.6	10.0 ± 5.6
36 h	23.3 ± 7.9	16.7 ± 6.9	20.0 ± 7.4

**Table 2:** Mean delayed mortality (% ± SE) of *Tenebrio molitor*, *Prostephanus truncatus*, and *Rhyzopertha dominica* maternal adult individuals exposed for 14 d to oat bran, maize, or wheat, respectively, after exposure to concrete treated with pirimiphos-methyl at control, 30 min, 3, 5, 8, 16, 24, and 36 h.

	Species		
Exposure	*Tenebrio molitor*	*Prostephanus truncatus*	*Rhyzopertha dominica*
Control	0.0 ± 0.0	0.0 ± 0.0	0.0 ± 0.0
35 min	0.0 ± 0.0	0.0 ± 0.0	0.0 ± 0.0
3 h	0.0 ± 0.0	0.0 ± 0.0	0.0 ± 0.0
5 h	0.0 ± 0.0	0.0 ± 0.0	0.0 ± 0.0
8 h	0.0 ± 0.0	0.0 ± 0.0	0.0 ± 0.0
16 h	0.0 ± 0.0	0.0 ± 0.0	0.0 ± 0.0
24 h	22.2 ± 8.2	18.5 ± 7.6	22.2 ± 8.2
36 h	30.4 ± 9.8	32.0 ± 9.5	29.2 ± 9.5

## Data Availability

Data are contained within the article.

## Supplementary Materials

The following supporting information can be downloaded at: https://www.mdpi.com/article/10.3390/insects14050430/s1, Table S1: The results of permutation test—*p* values for permutation tests (10,000 permutation rounds) for Procrustes distances among groups (exposed and non-exposed females and males) for each species separately. The statistically significant differences are labeled by red colored numbers and pink colored cells..
